# Hayek on complexity, uncertainty and pandemic response

**DOI:** 10.1007/s11138-020-00522-9

**Published:** 2020-07-29

**Authors:** Mark Pennington

**Affiliations:** Department of Political Economy King’s College, University of London, London, UK

**Keywords:** Hayek, Pandemics, Complexity, Uncertainty, Public policy, B00, B13, B25, B31, B53

## Abstract

This paper draws on Hayek’s distinction between simple and complex phenomena to understand the nature of the challenge facing policymakers in responding to the new coronavirus pandemic. It shows that while government action is justifiable there may be few systemic mechanisms that enable policymakers to distinguish better from worse policy responses, or to make such distinctions in sufficient time. It then argues that this may be a more general characteristic of large-scale public policy making procedures and illustrates the importance of returning to a market-based political economy at the earliest convenience.

## Introduction

The new coronavirus (Covid 19) pandemic that has engulfed the world in recent months has prompted unprecedented levels of government activism both in an attempt to control the virus and in attempts to curtail the economic damage arising from these policy measures. Governments across many parts of the world have effectively ‘closed down’ economic systems in response to the health threat posed by the pandemic. In some countries meanwhile governments have also committed themselves to policy measures - such as paying the wages of private sectors employees - that have rarely if ever been seen before.

This paper considers the challenge the pandemic presents to policy makers by drawing on F.A. Hayek’s distinction between simple and complex phenomena. It argues that while government action may be warranted, the complexities entailed in addressing the multiple socio-economic dimensions at stake mean that on many of these dimensions it may not be possible to discern the contours of an effective policy response. Or, if such responses can be identified, to secure their implementation in a sufficiently timely manner. The paper then considers the implications of Hayek’s perspective for broader socio-economic challenges that policymakers are increasingly being urged to assume, with a focus on post-pandemic risk planning and arguments for post-pandemic industrial policy.

The paper commences in section one by outlining Hayek’s distinction between simple and complex phenomena and how this is reflected in his critique of economic planning. It then proceeds in section two to consider some of the dimensions of complexity that underpin the coronavirus policy challenge. Section three argues that while government action may be a justifiable response to the pandemic, there may be few systemic mechanisms that enable policymakers to avoid large scale errors and to assess the effectiveness of alternative policy measures. Finally, section 4 argues that a continuation of activist government in a post-pandemic political economy may work to perpetuate aspects of the knowledge poor environment that characterises the pandemic itself.

## Hayek: Simple versus complex systems

A central aspect of Hayek’s social theory is the distinction he draws between simple and complex phenomena (for example Hayek 1967). Simple phenomena are those where it may be possible from a given starting position to predict the outcomes that will be generated by the application of a stimulus into a system. Scientific problems in some (though by no means all) parts of physics are of this type and they allow for the derivation of predictive, quantitative regularities by scientific analysts. Complex phenomena by contrast, refer to systems where the elements that make up a greater whole do not interact in a linear fashion and where the number of elements and the character of their interaction may be too vast for them to be comprehended by scientific observers. While non-linear systems can be scientifically modelled the relevant relationships may not typically be characterised with sufficient quantitative precision. The most that analysts may do when faced with complex phenomena is to try to understand the *general principles* that allow an order to form between the various elements– not to predict successfully the precise form that the order will take. All that science may be able to achieve in the face of such phenomena is to predict a statistical *range* of possible outcomes.

It is tempting to equate Hayek’s distinction between simple and complex phenomena with the difference between the subject matter of the natural and social sciences, but this would be mistaken (for a discussion see Caldwell 2004). While it is true that some natural sciences analyse simple systems, this is not always so. Many of the phenomena analysed in biology or ecology for example, are closer to complex systems. In such cases natural scientists may discover the general principles such as the principles of ecological succession that drive processes of environmental change. Typically, however, the scientists concerned may not be able to discern enough about the multiple contextual relationships between elements to predict successfully how eco-systems will evolve, given certain exogenous or endogenous changes to them (for example Botkin 1990; Scheiner and Willig 2011).

Turning to the socio-economic world, the challenge of scientific understanding relates to *even more complex systems*. Knowledge of all the varied and changing economic and cultural conditions that confront multitudes of people may not be comprehended by any social scientist or group of such scientists. Away from very basic forms of human society where the rhythms and routines of people might be observed and predicted with some accuracy by external observers – in more complex social orders the most that social science may achieve is an understanding of the general principles of human interaction and the broad patterns they produce. What may not be predicted successfully are specific responses to specific events. Economists may for example understand that if a good becomes scarcer in a market, various changes such as a rise in price and the search for new supplies or substitutes may be expected. They may not, however, predict successfully (other than by chance) what the specific responses will entail and the balance between the respective forces set in train (for a recent argument see Kay and King 2020).

Moreover, it may not even be possible to specify the range of likely responses because unlike the natural world the ‘human elements’ making up the social ecology are *creative* actors. On a Hayekian view, it is for this reason that the modelling of social interaction in terms of the predicted behaviour of statistically ‘representative agents’ that characterises contemporary neo-classical economics is of questionable value – for it is entrepreneurial *outliers* that drive processes of social change. While there may be ‘orderliness’ in society this does not equate to some static or fixed equilibrium state where change can only be accounted for through ‘exogenous shocks’ to an otherwise stable system. Rather, social interaction should be understood as process of *ceaseless* and sometimes turbulent change brought about by the generation of new ideas, processes, and ways of doing things (for example, Wagner 2020). This stance does not imply rejecting statistical modelling as a possible way to inform the day to day plans of individuals and organisations. Since future decisions grow incrementally out of past decisions there are *some* regularities that people can rely on to navigate their way in the world – though even here expectations will often be disappointed. Longer term econometric forecasting may however be of little use because the building of reliable econometric models requires knowledge of the factors that will shape the future in advance of their emergence (Parker and Stacey 1994: 76–77).

### Directive intelligence versus rules and decentralised feedback

The key point that Hayek takes from the distinction between simple and complex systems is that whereas the former can be subject to planning and control by a ‘directive intelligence’ the latter *cannot* be subject to such control owing to the overwhelming ‘knowledge problem’ that would face such an intelligence. Whatever objectives people may have, whether pertaining to health, economic growth, environmental protection, or some combination of these, will to a large extent have to be achieved via *indirect* mechanisms. Unless people choose a dramatically simpler form of social existence that might be more amenable to direction and control, then intelligent policy can only hope to find rules that allow for the many agents that make up complex social systems to adapt to the actions of others *without* having to know all of the factors that drive them. The mechanisms and rules that work most effectively in this regard will be those that provide relatively clear feedback to the agents and agencies concerned and enable relatively speedy adaptation to changing information about success and failure in reading situations at the local level.

In brief, this analysis underlies Hayek’s case for a market economy based on rules of private property and contract, over a planned or centrally managed economy. A market economy should be understood as a complex adaptive system where property rights and freedom of contract provide rules that enable an intricate ecology of dispersed individuals and organisations to experiment in responding to their own circumstances and for the results of these experiments to be communicated to neighbouring actors via profit and loss accounting and market price signals. The resultant ‘order’ is an ‘emergent’ property of the constant interaction between the various elements in a context where no ‘directive intelligence’ could be aware of all the possible and continually changing margins for adjustment.

Contrary to some critical readings of Hayek’s ideas (for example, Grossman and Stiglitz 1980), there is no suggestion that the relevant order in markets occurs instantaneously or that adaptations made are ‘perfect’. Rather, Hayek’s claim is a *comparative* one that market economies facilitate *more* learning and adjustment than would likely arise in a centrally managed alternative. Neither is there any suggestion that prices communicate all necessary knowledge. Hayek’s argument is the more modest one that market prices communicate in an indirect way *more* knowledge than would be possible without them. Price signals will always operate in a ‘noisy’ environment and given uncertainty about future states of the world entrepreneurs must try to understand whether shifts in prices reflect longer or shorter-term social trends and what the possible causes of these might be. Part of Hayek’s case for the market economy is that competition operates as a discovery procedure where different social constructions of what prices mean, are tested against one another and the relative strength of these subjective readings revealed through the account of profit and loss (for example, Hayek 1948; Hayek 1978a, b). Prices on this view are not ‘given data’ which are presented in their totality to actors. Rather they present themselves as overlapping ‘bits’ of data that both affect and are affected by a process of social contestation and interaction where widely dispersed interpretations of economic possibilities continually tussle with one another. Outside of such a rivalrous process the capacity to reveal the opportunity costs of alternative courses of action would be confined to the very limited imagination of a ‘directive intelligence’ or ‘social planner’.

It should be noted that none of these arguments are undermined by technological innovations such as for example the development of artificial intelligence or ‘big data’ gathering techniques which it is often suggested might allow for the replacement of market processes (for example Cottrell and Cockshott 1993; Phillips and Rozworski 2019 – for a critique see Hodgson 1998). On the one hand, these innovations increase the scope for decentralised agents -whether individuals, firms, or voluntary organisations, to increase the complexity of their own decision-making. This means that no matter how sophisticated the relevant technology becomes the complexity of the social system at the ‘meta-level’ will be higher than the cognitive capacities of any ‘directive intelligence’. Similarly, no matter how much data collection is facilitated by technology it will remain the case that the data at issue will not ‘speak for themselves’. Even if big data were to allow for a *retrodictive* understanding of the causes of socio-economic events this understanding will arrive ‘too late’ to act as a guide to effective decision-making. Moreover, to the extent that it provides an understanding of the past different people will interpret the *future* implications of the relevant data in different ways. On a Hayekian view, readings of data are no more than social constructions so the importance of competition in testing these different constructions against alternative readings remains paramount. Absent competition there is likely to be an increased risk of large scale decision failure where should those empowered to make decisions err in their choices then the effects are likely to be felt across the whole of society, rather than being confined to a relatively smaller portion of the community in question.

## The coronavirus pandemic as a complex problem

While the foregoing arguments are theoretical in nature, they are also empirically grounded claims that help to explain, *at least in a qualitative sense*, the superior performance of economies that rely on market processes relative to those that try to suppress them. Though Hayek develops a powerful case for the importance of market processes however, his perspective *does not* imply that there are market solutions to *all* socio-economic problems. In works such as *The Road to Serfdom* (1944) and *The Constitution of Liberty* (1960) Hayek sets out an extensive range of government measures where public goods style challenges arise. Dealing with infectious diseases such as the new coronavirus where these dynamics may be especially prevalent, may thus be a justifiable form of government action. Nonetheless, understanding and evaluating any such action will require proper appreciation of the levels of complexity in play and whether there are effective feedback mechanisms available to policymakers to cope with the uncertainties at hand. On a Hayekian view, the task is to find institutional configurations that allow for experimentation and feedback that is somewhat analogous to that provided by markets. With this challenge in mind this section sets out some of the complexities that may confront policymakers facing the coronavirus pandemic. The subsequent section turns to the existence or otherwise of mechanisms analogous to those in market processes that may enable decision-makers to avoid large scale errors and to learn from other decision-making nodes.

As with other pandemics, a first layer of complexity that policymakers must contend with in addressing the new coronavirus concerns the epidemiology of the virus itself. Epidemiological problems although involving natural science phenomena are not of the simple ‘physics’ type. While epidemiologists may discern the principles that govern how a virus spreads and perhaps simulate a range of possible outcomes –the precise manner of spread through a population will depend on a host of context specific variables that may not be accessible to the scientists or experts concerned. This challenge is seen most clearly perhaps in the problem of modelling the spread of the pandemic and the sometimes very different projections of the size and shape of the peak of the disease (for a discussion of this see Ormerod 2020). Will the pandemic peter out of its own accord without first having to affect a large percentage of the population? At what level of spread might ‘herd immunity’ be achieved? How is the spread of the virus affected by weather and geography? Will there be a second wave of infections? And will the virus mutate into a weaker or stronger form? Uncertainties surrounding answers to these and other such questions mean that modelling and data analysis will involve a good deal of subjective interpretation and scope for significant error.

An additional layer of complexity arises because the virus which is itself a complex phenomenon is interacting with a further complex phenomenon represented by the various political, economic, cultural and institutional arrangements across the world that might affect the manner of spread. As Ormerod (2020) notes, one of the key limitations of epidemiological models is that their projections often fail to account for human behaviour and changes in that behaviour – including those induced by public policy. There is, for example, considerable uncertainty about how the new coronavirus will respond to various public policy interventions. It is unclear how ‘lockdown’ policies might affect the size of a possible second wave of transmission. On one scenario lockdowns might be essential to reducing the spread of the disease to the point where infections in any ‘second wave’ could be more easily managed and controlled. On the other hand, however, it could be that the success of lockdowns in limiting the spread of a first wave of the virus will only contribute to a much larger and potentially uncontrollable second wave of infections owing to the limited extent of herd immunity arising from the lockdown measures.

Still further complexity is injected into the policy conundrum by uncertainties about how different populations with different social attitudes, time horizons and belief systems may respond to the various policy measures that are adopted, or to news about developments that are affecting the spread of the virus. Within this context, there is a distinct possibility for ‘Lucas effects’ to arise. In macro-economic analysis these refer to situations where public policy measures might be counteracted by shifts in behaviour which are a response to the measures concerned (for example, Lucas 1976). If for example policymakers seek to raise inflation in the hope of lowering unemployment, then this may lead to a shift in employers inflation expectations which may lead them to *decrease* employment. Macro-economic models if they are to be useful to policymakers need, therefore, to factor in how changes in policy might change the expectations and behaviour of the agents on which the policy is supposed to act. In the specific context of pandemic response it is possible that if people come to believe that a vaccine is imminent or that herd immunity is close to being achieved they may start to behave in ways – such as abandoning social distancing measures – that make the immediate problem worse**.** On a Hayekian view, however, there is a significant ‘knowledge problem’ for policymakers in understanding *how* expectations will be changed by the pattern of events or by policy interventions – and this problem is especially severe in a context of heterogenous individuals with divergent ideas whose actions cannot be reduced to those of a ‘representative agent’.

The complexities discussed thus far raise significant challenges for policymakers even if they concern themselves solely with managing the health effects of the virus. To approach pandemic response in such a way would be to see it as primarily a ‘technological problem’ – where the task at hand is to allocate resources to achieve a singular ‘technical’ end. Pandemic response, however, is not best understood as a narrowly technical exercise but as an ‘economic problem’ which involves allocating resources between multiple competing ends. In the context of health objectives there are complex and uncertain trade-offs to be made between the possible reduction of deaths from the coronavirus that might follow lock-down measures and the possible increased deaths arising from illnesses that might go undiagnosed or untreated because of such measures**.** Away from the problem of trying to trade off deaths from these different sources, there are also costs to be considered pertaining to the possible deterioration in people’s mental health as well as to possibilities such as increases in domestic violence that may also accompany prolonged periods of confinement and social isolation**.**

Turning away from these health-related trade-offs to broader socio-economic questions there is great uncertainty over the extent to which the socio-economic damage that may have been inflicted by a less controlled spread of the virus is matched or outweighed by the scale of the socio-economic costs associated with the measures being taken to contain it. Part of this challenge can be understood as arising from trade-offs between different sources of what Higgs (1997) refers to as ‘regime uncertainty’. On a Hayekian view, one of the primary functions of social institutions such as private property rights and contract law is to provide a measure of certainty to decision-makers in an otherwise highly complex and uncertain world. Agents operating in a market economy cannot be sure that their *ex ante* assessment of a decision situation will be confirmed *ex post* – but secure property rights do provide some degree of certainty that agents can retain the profits from decisions that turn out well and face the costs of those that turn out badly. Regime uncertainty however arises precisely in contexts where the basis of social rules such as security of property *itself* becomes subject to uncertainty.

In the context of pandemics regime uncertainty could arise from the failure of political authorities to act. An uncontrolled pandemic might lead to social dislocation and a break down in respect for the social norms and institutions – such as respect for property – that provide much of the glue sustaining social life. On the other hand, however, regime uncertainty might also arise from badly judged public policy interventions. There is a danger that political authorities may intervene in market relationships in unpredictable ways to a point that this destroys the confidence of people in the impartiality of the law. Consider in this context the worker ‘furlough schemes’ that have been introduced in parts of Europe and the United States. These schemes pay out a large proportion of the wage bill for employers in businesses that have been closed by lockdown policies. In a context where public health measures have effectively ‘paused’ the economic system there is a strong case for schemes of this kind. There is however also a danger that political pressures could be brought to bear on policymakers in ways that introduce significant arbitrariness in the operation of the schemes. The problem here is that any evidence of political favouritism may reduce the long term confidence of investors who in addition to navigating the inevitable uncertainties of business life may also fear having to anticipate potentially arbitrary interventions that favour some sectors over others. A different though related set of issues arise with the introduction of measures such as contact tracing schemes where there are concerns that personal data initially used to track those infected with the coronavirus might be used by political authorities as sources of information to be used against their opponents.

Away from these issues of regime uncertainty there are wider and equally complex challenges arising from attempts to manage the macro-economic consequences of the pandemic and the policies to control it. Returning to the case of employment ‘furloughs’, while government spending may be necessary to support workers and employers in the immediate term there is also a danger that schemes of this kind may cause lasting economic damage if they are maintained for too long. If the effect of lock-down policies is to induce longer term changes in behaviour which may persist after the pandemic has passed – such as a greater reliance on home working, reduced demand for office space or increasing popularity for online delivery in fields such as education - then these changes may imply the need for significant economic restructuring and reallocations of labour and capital which an overly long furlough period would delay. Such changes cannot occur instantaneously but must occur *over time* as entrepreneurs try to discover which combinations of labour and capital are best suited to the new circumstances – and while this process unfolds there will inevitably be ‘unused capacity’ as a proportion of labour and capital will be left unemployed or ‘idle’. In these circumstances, Keynesian inspired ‘stimulus packages’ and macro-economic interventions that seek to ‘close the output gap’ and to preserve the *pre-pandemic* pattern of employment by maintaining ‘aggregate demand’, are subject to the concerns raised by the Hayekian critique of such measures in the context of financial or other economic crises (for a recent statement of these see White 2014). More specifically, a focus on aggregate demand may obscure underlying shifts in the structure and distribution of demand across different sectors and hence the need for relative price adjustments to signal the need for changes in the structure of production. Large scale public spending programmes that are not themselves subject to profit and loss signals risk delaying the necessary adjustments and indeed may lead to malinvestments. Judging the timing for withdrawing furlough measures or macro-economic stimulus policies that seek to preserve pre-pandemic employment patterns will therefore be subject to a high level of both economic and political uncertainty and modelling efforts to time these decisions and their likely effects will involve a high degree of subjectivity and potential for significant error.

## Complexity and pandemic response: Avoiding systemic error and the scope for policy learning

On a Hayekian view, understanding society as a set of ordered though dynamic relationships rather than a static equilibrium means that justifying public policy responses to the complex dilemmas set out in the previous section *does not* require that these responses will be ‘optimal’ - precisely because the uncertainties and complexities at hand may preclude the identification of ‘optimal solutions.’ Given the character of pandemics as public health problems involving potentially significant externalities, market solutions and those based on voluntary associations may not be viable^1^ so there are strong grounds for endorsing some form of public policy response (for example, Ramanan and Malani 2011). Nonetheless, the question that Hayek’s perspective might ask is whether there are any *systemic* mechanisms that may enable policy-makers to avoid large scale decision-making errors and to identify and act upon knowledge of *relatively* ‘better’ or ‘worse’ responses.

### Avoiding Systemic Error

Turning first to the question of avoiding large scale error, it is important to recognise that as Lindblom and other analysts of public administration have shown, states are *not* unitary hierarchies operating with a singular ‘directive intelligence’, but are better understood as networked structures where many different organisational ‘brains’ are linked together through bargaining and mutual adjustment (Lindblom 1965, 1979). Unlike most market settings however, these are networked arrangements where competition is limited and where particular organisational nodes, or coalitions of actors may exercise a *disproportionate or monopolistic* influence over the network, as a whole (Wagner 2016, 2020). In the specific case of pandemic response public health bodies and economic agencies such as treasuries and central banks are ‘big players’ within the social ecology that owing to their size and/or unique powers, have the potential to inflict large scale errors on the wider polity (Koppl 2002). It should be acknowledged here that expert epidemiological opinion and economic opinion over how to respond to the pandemic is *not* characterised by a consensus so in practice the decisions made by public health authorities, treasuries and politicians will reflect those of a *dominant coalition* in the jurisdictions concerned.

Within this context, an important institutional limitation on the scope for systemic error by ‘big players’ is that the world lacks an international administrative structure with the powers to enforce a ‘global governance solution’ to the pandemic. Relative to what might be the case under the existing more fractured and decentralised governance regime a global governance approach might increase the likelihood of a large scale health or economic disaster should those who control public health bodies, treasuries and central banks err in their choice of policy measures. Writing in the context of common pool resource management Elinor Ostrom makes a point that may be equally applicable to pandemic response.

“Where there is only a single governing authority, policy-makers have to experiment simultaneously with all the common pool resources within their jurisdiction with each policy change.... Thus an experiment that is based on erroneous data about one key structural variable or one false assumption about how actors will react can lead to a very large disaster.... The important point is that if systems are relatively separable, allocating responsibility for experimenting with rules will not avoid failure but will drastically reduce the probability of immense failures for an entire region (Ostrom 2006: 284).”

The current pattern of pandemic response does not rely heavily on global ‘big-players’ or a ‘single governing authority’. Rather, there is some level of decentralisation exercised largely through the powers of nation states and to a lesser extent *within* nation states where federal political systems are operative. It is perhaps significant in this regard that many countries started to introduce response measures, such as social distancing and travel restrictions, *before* international bodies such as the World Health Organisation made such recommendations, while others resisted implementing measures after the WHO declared a ‘global pandemic’. Similarly, it may be significant that in federal political systems such as the United States, some jurisdictions introduced lockdown measures before such recommendations were made by the federal Centre for Disease Control – as did many private employers, while other states resisted these calls.^2^

Nonetheless, it needs to be emphasised that while the relatively decentralised structures of international and in some cases national governance may reduce the risk of large-scale policy failure, the powers exercised by public health and economic agencies *within* nation states are such that the scope for error by ‘big players’ remains considerable. The problem here is that there a few mechanisms that enable an effective balance to be struck between the need to reduce the high transactions costs that may be associated with an excessively decentralised response to the virus with the systemic risks associated with over-centralisation. As Coase (1992) points out, in market economies under normal circumstances the balance between centralisation and decentralisation is continually determined and re-determined through an ongoing process of competition embedded in profit and loss signals as firms of different sizes compete within one another and the process of mergers, demergers and new entrants unfolds. Similarly, Ostrom points to the importance of institutional diversity in addressing problems of scale where the existence of competing and overlapping decision centres whether private, communal or public allow multiple comparisons between governance regimes and knowledge of relative successes and failures in the supply and management of public goods to be spread through an experimental process (for example, Ostrom 2006). This may be particularly important in the context of pandemic response because the management challenge at hand may not equate to a *singular* collective action problem. Rather it may be that the spread of the virus requires responses to a range of multiple and more localised collective action problems the structure of which will vary according to geography, population density and the cultural characteristics and beliefs of the populations concerned. Unfortunately, however, there may *not* be mechanisms to decipher what level of political and legislative decentralisation best matches these varying socio-environmental characteristics.

This is not to say that there is *no* scope for determining such a balance. Centralised trial and error and ‘muddling through’ by ‘big-players’ can lead to *some* changes in decision-making that reduce the scale of individual public decision failures. In both the United Kingdom and the United States for example, the initial failure of national public health agencies to involve private and voluntary sector agents in the roll out of coronavirus testing appears to have been a key factor behind what was initially a miniscule number of completed tests. Subsequent lobbying by private bodies and media commentary led to a reversal of this stance which was then followed by significant expansions in testing delivery (BBC News May 02, 2020).

Pointing to the success of centralised trial and error in such examples, however, may only serve to illustrate the broader weaknesses of such an approach when faced with more complex, multi-dimensional challenges. In the example just given, the focus of trial and error learning was confined to a very narrow ‘technical’ end – i.e. how to increase the number of completed tests. Problems of this kind may be more amenable to a centralised learning process because there is scope for ‘before and after’ type observations to discern the effect of particular interventions. Learning of this kind may be much more problematic however, when the problems at hand are ‘economic’ in nature and involve many competing margins for adjustment that may only be revealed in a context where multiple governance models can coexist and where trial and error learning occurs in a more decentralised manner. With respect to broader matters of pandemic response therefore, such as the costs of decisions to lock down populations and businesses or to engage in economic policy measures to counteract the effects of such decisions, then the scope for institutional evolution may be very limited. Owing to the ‘emergency’ nature of the pandemic, to all intents and purposes the inhabitants of nations may be ‘stuck’ with whatever institutional configurations they have. This may be a significant problem, because institutional configurations that work well under ‘normal’ circumstances may not be well adapted for pandemic response and the scope to avoid potentially large-scale decision failures associated with these may thus be limited.

### The scope for policy learning

While the avoidance of large-scale policy failure is an important institutional consideration, from a Hayekian view so too is the importance of generating counterfactuals to allow for policy-learning. As with the previous discussion this speaks to the value of a fractured governance regime based on nation states or varying degrees of federalism rather than a global governance approach. Just as central economic planning deprives consumers and producers of the information generated by competitive experimentation in a market, so a global governance approach would deprive decision-makers of any sense of the possible opportunity costs associated with different responses to the virus. On this understanding, it may be a virtue of the predominantly nation-centric approach that while many countries have chosen to pursue ‘lockdown’ measures, others such as Sweden have opted for a very different approach based on allowing a gradual spread of the virus through the population. The Swedish approach may not be the ‘right one’ but without its existence or others like it there would be no comparative base against which to evaluate the policy measures adopted elsewhere, and potentially to learn from these. Indeed, outside a context that allows for at least some level of decentralisation terms such as policy ‘success’ or ‘failure’ are meaningless. On a Hayekian view, one cannot judge policy responses against a vision of an ‘idealised’ policy that might be implemented by omniscient agents because in conditions of complexity and uncertainty knowledge of such an ideal is elusive. The success or failure of a policy can *only* be determined through a process of *endogenous comparison* – and this requires a framework that allows for the generation of such comparisons.

While the scope for counterfactuals generated by the fractured nature of international governance is important for policy learning, in the context of pandemic response it needs to be recognised that politicians and regulators in nation states and other levels of decision-making face a significant ‘signal extraction problem’ in deciphering what the results of various policy experiments may mean and whether any lessons can be applied elsewhere. As noted earlier, the prices, profits and losses generated in markets do not necessarily provide a transparent signal ‘telling’ decision-makers how to act. In market settings, however, the feedback received from consumer spending decisions on specific products is such that the profits/losses made relative to those of competitors, combined perhaps with knowledge from market research, provides a *relatively* clear signal that competitors should move in the direction of their rivals practices. Relative to this situation the ‘noise’ around the different outcomes arising from various approaches to managing the coronavirus may be much more pronounced. Thus, even if the Swedish model produces satisfactory results from a Swedish perspective it *does not* follow that the same results would or could be achieved with this model in countries such as the UK or France that have very different cultural traditions with which the policy would be interacting. Similarly, it is hard to judge whether the apparent success of countries such as Germany and Switzerland in having a death rate far lower than that found in the UK follows from the characteristics of the populations infected by the disease, other factors such as housing conditions or the nature of urban form in these countries or, whether the lower death rates reflect instead the superiority of health care systems that make much greater use of private providers and market forces.^3^ If any of these factors are significant, then it is far from clear that any policy lesson from the pandemic could be implemented with sufficient speed, or indeed whether it could be implemented at all.

The difficulty of interpreting policy results highlighted above is compounded by the question of what an appropriate time frame might be in which to make a comparative evaluation of the health and socio-economic effects of alternative policy measures. Will countries that look to be performing relatively well with respect to death rates look to have chosen effective responses if a second and possibly larger wave of the virus arrives in the autumn/winter when the prevalence of some level of herd immunity might be desirable? With respect to economic evaluation, will countries that have adopted measures that have in the short term succeeded in reducing the spread of the virus by massively curtailing economic activity be able to sustain such measures if the virus remains a public health hazard over a number of years? Given the complications that time frames introduce into the analysis it is hard to see how any policy lessons if they can be detected might be adopted with sufficient speed. Moreover, it should be noted here that any short to medium term evaluations may change radically depending on whether an effective vaccine is found. Lockdown measures that may have saved lives at great economic cost might still look to have been the best available response should a vaccine be developed relatively speedily. If a vaccine is not forthcoming, however, then those responses or non-responses that have not involved large scale economic contraction may turn out to be the most effective with respect to health and socio-economic objectives.

Finally, it should be emphasised that while the possible arrival of a vaccine may be affected by investments made by public and private agents, no matter how much private or public money is spent in this regard the discovery of a vaccine will to a significant degree lie beyond *any* authorities control.

### Pandemic response as muddling through

The conclusion that would seem to follow from this analysis is that the scope for both error avoidance and policy learning in the context of pandemic response is heavily constrained. The rationalistic models depicted in many public health and welfare economics accounts that conceptualise policy as a process wherein policymakers list the possible options they face, evaluate the options, and then select the option that generates the highest value are entirely inappropriate to this and indeed most other forms of government decision-making. In the specific case of pandemic response, the level of complexity and uncertainty may be so great that it is not possible for such calculations to be made. The data that would be needed to calculate in this manner either do not exist – at least not in ‘concentrated or integrated form’ (Hayek 1945: 520) and, to the extent that there are institutional procedures that help to generate and communicate relevant data the relations between cause and effect and the micro-level connections that underlie such data are opaque. Much of the response will therefore be based on centralised guesswork by ‘big players’ and while there may be no alternative other than for policymakers to rely on subjective interpretations of epidemiological and economic models to guide their decisions, these are fraught with the possibility of large scale error.

Now of course, scientific understanding in both the natural and social world is always highly imperfect and as new data emerge this may enable an evaluation of which models were more accurate in an *ex post* sense. The Hayekian perspective presented here is not incompatible with this stance but it emphasises that should it be possible to explain retrospectively which policy responses have been more or less efficacious this will not necessarily inform policy-makers whether the same responses would work for a future such event. Beyond perhaps some very basic and general lessons such as the importance of maintaining adaptable/flexible health care systems (which are desirable at all times), some (though not all) social distancing measures, and perhaps the wearing of face coverings it may be hard to discern what lessons should be learned from the current episode. This does not necessarily undermine the case for a public policy response – though neither does it imply that voluntary measures are obviously inferior - but at the very least it suggests that expectations for publicly organised pandemic response should be modest. Though it is not a conclusion that many citizens, politicians or social scientists may feel comfortable in accepting, the Hayekian perspective suggests that policymakers may be operating in a fog of ignorance where insofar as tolerable responses are reached, these may to a large degree result from fortuitous accidents arising from a process of ‘muddling through’.

## Conclusion: Implications for a post-pandemic political economy

The analysis thus far has focussed on the challenge of discerning appropriate responses to the pandemic, but the Hayekian perspective also points to important considerations for the post-pandemic world. If historical experience of crises whether wars or natural disasters are any guide to the post-pandemic political economy, then this period seems likely to be characterised by increasing calls for more government activism and control (on this see Higgs 1987). On the one hand, these calls may be driven by demands for preventive measures to avoid anything like the present crisis happening again. On the other hand, states that have assumed significant control over resource allocation during the pandemic may be reluctant to relinquish their powers and may be encouraged to retain them by those who envisage significantly expanding the role of government in the economy. The concluding part of this paper briefly sets out why these forces should be questioned.

### The conceit of post-pandemic risk planning

While it is understandable that citizens and politicians should seek to avoid a repeat of current events, the Hayekian perspective suggests that ‘scientific management’ of future risks is unlikely to be successful. That politicians and regulators were, prior to the current pandemic, overwhelmingly concerned with the threat of a ‘climate emergency’ and seem to have been taken aback by the new coronavirus, only serves to demonstrate that there is great uncertainty over which risks should be the focus of attention. Neither are such oversights confined to governmental actors – as recently as January this year a variety of corporate bodies representing major business interests at the World Economic Forum were also citing climate change as the most important global risk, with the threat of infectious diseases rating much lower in the scale of priorities (World Economic Forum 2020). To point out these oversights is *no*t of course, to say that climate change or other such risks should not be taken seriously. Rather, it is to highlight the problem of assigning weightings to these risks in conditions of radical uncertainty.

Looking to the future, it is not the case that precautionary measures should be taken against *all* possible catastrophes because the accumulated costs of responding to every such possibility may be as great, or greater than that of the catastrophes to be avoided (on this see for example, Martin and Pindyck 2015). It is, therefore, essential to choose from multiple conceivable disaster avoidance measures, which should be prioritised. Should the focus of risk avoidance be on the possibility of further pandemics, climate change, the threat of nuclear terrorism, or bioterrorism? The problem here is that many of the parameters relevant to discerning these probabilities and the possible interactions between them are simply unknown. This challenge is surely significant in the context of known threats, but it is compounded by the possibility of unknown, unknowns. In conditions of radical uncertainty, it is not merely that actors may not know which possibility from a given set will occur but that the set itself may be unbounded and hence unknowable (Knight 1921).

None of the above should be taken to imply that all scenario planning and spending based on such planning to account for future risks should be discarded. There is a *limited*, prudential case for private and public funding of measures to guard against future pandemics or other threats such as climate change. What the Hayekian perspective suggests however, is that relatively little faith should be placed in these measures because given the nature of uncertainty, the next disaster to strike may well be one that has yet to be conceived. In the final analysis, ‘what cannot be known, cannot be planned for’ (Hayek 1988) so there are grounds to be wary of granting authority to political agencies that justify their assumption of new powers on the basis of highly uncertain and perhaps unknowable likelihoods of future risks. Moreover, insofar as authority is granted to public bodies rather than for example leaving these decisions to private insurance markets, then an important implication from the Hayekian analysis is that this authority should be fractured and should where possible avoid reliance on global governance schemes. Should the wrong risks be chosen by big players in a global governance structure then the negative consequences will be felt globally. In a more fractured regime on the other hand, unless all decision-making units perceive risks in the same way then the greater heterogeneity of decisions, may reduce though it may not eliminate, the possibility of system-wide failures of risk management.

### Growth, resilience, and the conceit of the transformational state

If there is reason to doubt the efficacy of centralised governance in strategic risk planning then the most effective and multipurpose ‘insurance policy’ that might account for the broadest range of future risks may be to sustain robust levels of economic growth. The resources generated by such growth may provide *resilience* against risks from *multiple* directions. In a context where states have recently assumed massive responsibilities for directing economic activity, however, many may argue that securing growth and the form it will take should be the responsibility of the state. There is a long line of thinking in the social democratic and progressive traditions, inspired by thinkers such as John Dewey (1927) and John Maynard Keynes (1931) and reflected more recently by Mariana Mazzucato (2013), which suggests that crisis situations require bold and radical experimentation by state agencies. On this view, only the state has the capacity to engage in the ‘transformational’ measures that might be required to ‘jolt’ society out of crises events. Support for this worldview was evident prior to the current pandemic with the renewed enthusiasm across the political spectrum for various industrial policies, green ‘new deals’ and targeted protectionism, but post-pandemic these pressures may well grow in intensity (for example IIPP 2019).

On a Hayekian view, however, these trends should be resisted. One reason for this is the danger of injecting a further source of ‘regime uncertainty’ into what may already be unstable political economic environments. In responding to the pandemic governments across many parts of the world have already engaged in socio-economic interventions of unprecedented scale, and as noted earlier both the socio-economic effects of these interventions and of measures to unwind them are subject to significant uncertainty and scope for systemic error. These policies have also followed in the wake of the massive interventions in financial markets by monetary authorities in the 12 years since the crisis of 2008.^4^ To embark on ‘transformational’ measures in such a context would be to generate an additional source of systemic risk and may further undermine the background foundations of competition and private contracting, in favour of a regime where discretionary political and bureaucratic power by ‘big players’ exercises a growing and unpredictable element in the calculations that private agents must make.

The second and related reason to resist any post-pandemic expansion in governmental power is that this may *recreate* the severe knowledge problems that politicians have faced in choosing how to respond to the pandemic *- on a near permanent basis.* Now, this is a bold conjecture, so it is important to be precise in specifying the content of the claim.

Part of the knowledge problem of pandemic response discussed in this paper is that the *emergency* nature of the situation severely limits the scope for policymakers to avoid large scale errors and to learn in a sufficiently timely manner. This is clearly *not* a characteristic that would be shared by measures such as industrial policies that can be implemented over a longer time scale and with greater scope for trial and error learning. Nonetheless, from a Hayekian perspective, the nature of this learning will be dominated by the ‘muddling through’ of ‘big players’ in a context that limits the generation of counterfactuals and which operates largely *without* guidance from competitive profit and loss signals. It is in this respect that ‘transformational’ public policies may replicate the knowledge poor environment that characterises the setting of pandemic response.

Compared to the decentralised experimentation that takes place in competitive markets the type of experimentation involved in state-centred schemes of economic ‘transformation’ lacks a systemic mechanism to decipher relatively better from relatively worse decisions. It is the systemic discipline provided by competition and profit and loss accounting that, while never guaranteeing successful investment or effective coordination, *increases the chance* of discovering beneficial investments and the shutting down of those that fail to add value. The greater pluralism of decision-making in markets compared to democratic or bureaucratic settings -the fact that in most markets multiple firms offer consumers different products and services - facilitates comparisons between alternatives. Moreover, in these settings there is no need for agents to fully comprehend the reasons underlying their success or failure. The generation of profits and losses, combined with the existence of hard budget constraints, continually prods agents towards better decisions and away from relatively worse ones *without* needing to wait for complex factual and theoretical knowledge or the interpretation of data regarding cause-effect relationships.

By contrast, state-dominated ‘transformations’ thwart the emergence of counterfactuals and knowledge of opportunity costs by limiting competition. As with pandemic response, the scale of the expenditures or the scope of the regulations concerned, and the lack of profit and loss signals attached to them, may block the communication of which ‘bits’ of expenditure or regulation are adding value. Without profit and loss accounting policymakers must rely on centralised guesswork, or at best reliance on modelling procedures that if they ever do generate knowledge of cause-effect relationships may not do so with sufficient speed to shut down failing projects. This problem is compounded by the much softer budget constraints in the public sector that may enable policymakers to continue supporting ventures that fail to add value. Even when particular firms or industries do make positive returns, if these have been supported by public funds then knowledge of the returns that might have been generated had tax payers been allowed to invest the capital elsewhere *will be foregone*. To the extent that ‘transformational expenditures’ work to promote growth, therefore, then as is the case with pandemic response, this may to large degree be the result of *fortuitous accident*.

To illustrate the empirical relevance of this perspective, consider the arguments for industrial policy espoused recently by Mazzucato (2013). She maintains that because some of today’s technological innovations had their origins in acts of government spending rather than private investments this demonstrates that ‘directional planning’ by the state can improve on market outcomes and that states should be bold in their willingness to spend on transformational projects. Yet the evidence Mazzucato cites simply fails to support these conclusions (see for example, Mingardi 2015). First, she ignores the opportunity costs of the massive cold war related military spending she claims was partly responsible for innovation and growth. While some of this expenditure may well have added value Mazzucato offers no analysis of the multiple acts of military spending that *failed* to stimulate beneficial innovation, and which were not shut down. Second, Mazzucato fails to recognise that those elements of public spending that may have generated benefits were not ‘planned’. At no point does she specify processes that demonstrate how ‘directional intelligence’ and ‘strategic planning’ led to specific instances of success. On the contrary, the success stories she refers to – such as the development of various digital technologies, appear to have been *unintended or accidental consequences* emerging from essentially random spending in the defence sector – unintended consequences that were adapted to and seized upon by private agents operating in competitive markets guided by profit and loss signals (ibid).

Mazzucato is not alone in downplaying or ignoring opportunity costs and in failing to specify the processes that allow the ‘directive intelligence’ of the state to ‘beat the market’. A similar problem besets the strands of political economy literature that favour targeted protectionism (for example Chang 2007). In this instance, the relative success of *some* countries that have pursued protectionist policies is touted as evidence in their favour without adequate discussion of the multiple examples where similar measures have failed to deliver success. Moreover, where successes have occurred little if any account is given of the processes or mechanisms that could credibly connect the outcomes to specific acts of directive intelligence. It is simply assumed that the intervention in question was responsible for the outcome rather than arising despite the intervention, or as an accidental or unintended consequence from it (Panagariya 2019).

The Hayekian perspective that has informed this paper does not claim that it is *impossible* for industrial policies, large public spending projects or targeted protectionism to generate positive results, any more than it claims that is impossible for governments to respond effectively to pandemics. As with pandemic response, what it suggests is that relative to markets there are few *systemic mechanisms* that enable decision-makers to learn whether their decisions add more, or less to public welfare than possible alternatives. From a Hayekian perspective, what the Dewey/Keynes/Mazzucato case for state-based experimentation amounts to is the suggestion that if governments commit to spending enough public money on their favoured projects it would be remarkable if none of this expenditure did any good. Yet, this position hardly amounts to an endorsement of the transformational potential of the state. That it may be necessary to rely on the ‘muddling through’ of public agencies when responding to a pandemic, does not imply continued deference to such agencies when the emergency has passed.

## Footnotes

1 This does not mean that markets should be curtailed in those sectors which have not been shut down by the various emergency measures adopted by governments. On a Hayekian view there is, for example, good reason to believe that the introduction of price controls when concerns about impending shortages arising from government measures are at their height should be avoided – since such controls stifle the market signalling of which goods are becoming more or less scarce.

2 The World Health Organisation declared a ‘global pandemic’ on March 10th. The United States introduced travel restrictions on China in late January. California was one of the first states to introduce ‘lockdown’ policies while South Dakota, for example, continually resisted these – on the latter see *The Guardian *2020.

3 As of late May 2020, the increase in the total number of recorded deaths per million in the United Kingdom since the onset of the pandemic was several multiples of those recorded in Germany and Switzerland – see *Financial Times*, May 28th, 2020. The latter countries have health care models which combine private and social insurance with a preponderance of competitive private delivery. The UK health care system by contrast is a publicly funded model with a near public monopoly on the supply and delivery of health care services.

4 For an argument that the crisis of 2008 may itself have reflected a systemic epistemic failure arising from excessive centralisation of financial regulation rather than arising primarily from ‘market failures’ see Friedman and Krauss (2011).
